# Supplementary material for: Label-Free Sensing of Cell Viability Using a Low-Cost Impedance Cytometry Device
Source: Micromachines (Basel). 2023 Feb 9;14(2):407. doi: 10.3390/mi14020407 (PMC9963508; doi:10.3390/mi14020407)
Supplement: Supplementary file 1 [file micromachines-14-00407-s001.zip › micromachines-2187885-supplementary.pdf]

Label-free sensing of cell viability using low-cost impedance cytometry devices

Bowen Yang [1], Chao Wang [1] , Xinyi Liang [2] , Jinchao Li [1], Shanshan Li [1,3,*] , Jie Jayne Wu [4,*] , Tanbin Su [1] and Junwei Li [2,*]

1 Hebei Key Laboratory of Smart Sensing and Human-robot Interactions, School of Mechanical Engineering, Hebei University of Technology, Tianjin 300130, China

2 Institute of Biophysics, School of Health Sciences and Biomedical Engineering, Hebei University of Technology, Tianjin 300401, China

3 State Key Laboratory of Reliability and Intelligence of Electrical Equipment, Hebei University of Technology, Tianjin 300132, China

4 Department of Electrical Engineering and Computer Science, The University of Tennessee, Knoxville, TN 37919, US

* Correspondence: sli_mems@hebut.edu.cn (S.Li.) jaynewu@utk.edu (J.J.Wu) ; junwei_li@hebut.edu.cn (JW. Li)

1. Typical signals from 10μm-scale Au electrodes and 100 μm-scale ITO electrodes

Figure S1 provides the typical differential signal from two kinds of double-differential electrodes. Among them, the plot in Figure S1(a) was recorded by 10μm-scale Au electrodes, as reported in the previous literature. The plot in Figure S1(b) was recorded by 100 μm-scale ITO electrodes fabricated by the low-cost microfabrication method. Both of them show a bipolar double gaussian distribution waveform. Although the amplitude did not consistent with each other exactly, the data can be applied without any loss of accuracy. The reason lies in the method of data analysis described in section 3.

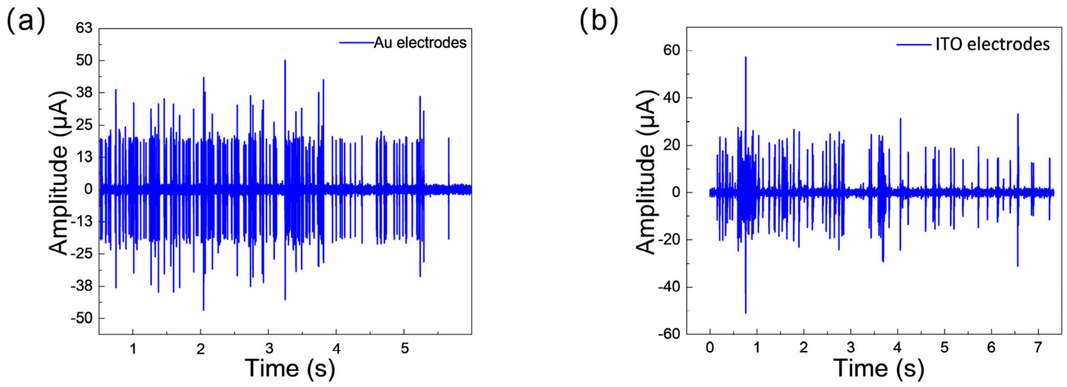

Figure S1. Comparison of typical signal from 10μm-scale Au electrodes and 100 μm-scale ITO electrodes. (a) differential current from 10μm-scale Au electrodes. (b) differential current from 100μm-scale ITO electrodes.

2. Data analysis and position calibration of the double differential signal

From the previous work [1], we can get the signal differential current generated by double differential impedance cytometry device as shown in Figure S1. When a particle passes through the center of the microchannel from left to right along with the channel distance, it first passes through the electrode with opposite phase angle voltage, and then reaches the GND electrode through the floating electrode. The differential current signal presents a double-peak shape due to the non-homogeneous electric field distribution by the floating electrode. As the particle transit through the GND electrode to the central electrode, an opposite peak appears with a much higher current amplitude compared to the previous signal. Because the electric field intensity is much higher than before. Then the particles pass through the symmetrical electrode, and the signal is symmetrical to the front. The small double peak in the signal is the particle position calibration signal.

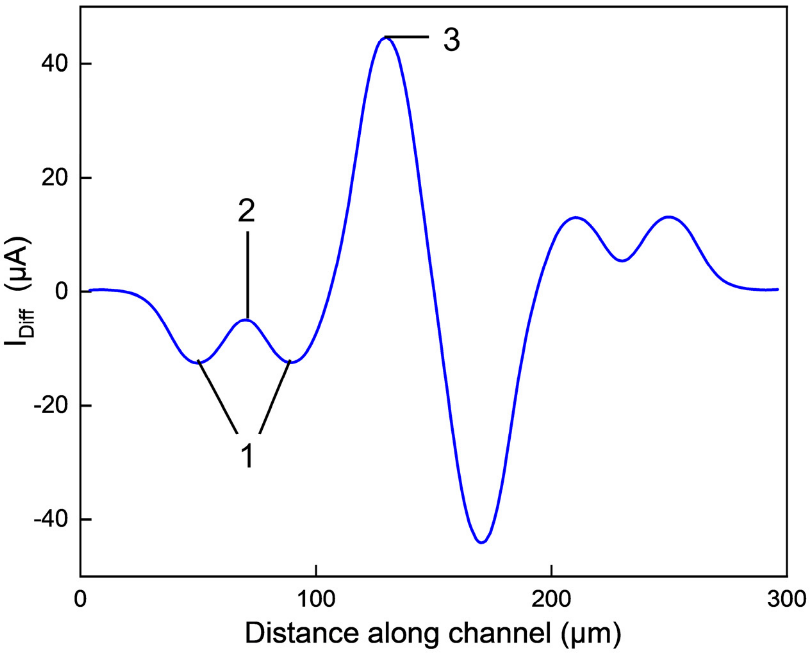

Figure S2. The signal of the double differential impedance cytometry microfluidic. Number 1 represent the height of the double-peak, 2 represent the height of the valley, 3 represent the high of major peak.

We use P represents the height of the double-peak (S1) and p represents the height of the valley (S2). α is defined as the factor of the particle position calibration. α is a real number between 0 and 1, which is defined:

$$\alpha = \frac{P - p}{P} \tag{S1}$$

The higher peaks (3) in signal are defined as raw current amplitude(A), the raw electrical diameter (RED) is adopted from previous studies:

$$RED = GA^{\frac{1}{3}} \tag{S2}$$

Where G is the geometric constant depending on the channel size (G=2.15 μm μA^{-1}, calibration by 7 μm beads). Because the electric field intensity generated by the coplanar electrode is non-homogeneous, when the same particles flow through the channel at different heights, the

current amplitude may be different. For example, the current amplitude of particles flowing close to the electrode will be larger, while the current amplitude of particles flowing far away from the electrode will be smaller. Therefore, the raw electric diameter has error in particle measurement, and the previous position factor needs to be used for calibration. First, normalize the data. The normalization is defined as:

$$NED = \frac{RED}{Bead\ Size} \tag{S3}$$

Where RED is the raw electrical diameter measured by the MIC ship. NED is the normalized electrical diameter. Bead size is the size of the calibration beads. The scattered data obtained from the experiment can be fitted into a linear function:

$$NED = c_1 \times \alpha + c_2 \tag{S4}$$

Where c_1 and c_2 are the calibration factors used to calculate the calibration electrical diameter. Since linear fitting is adopted, the fitting parameter c_1 and c_2 can be universally applicable to particles with different size by the same MIC ship. The electric diameter after calibration can be obtained from:

$$CED = \frac{RED}{c_1 \times \alpha + c_2} \tag{S5}$$

Where CED is the calibrated electrical diameter.

3. Comparisons of our device with representative devices in previous studies as shown in Table S1

Table S1. Comparisons of our device with representative devices in previous studies

Electrodes width	Electrodes material	Number of electrodes	Ref.
100 μm	ITO	7	Our work
20 μm	Au	3	[2]
30 μm	ITO	4	[3]
10 μm	Au	7	[1]

4. The cost breakdown of our device

Table S2. Cost breakdown of our device

Glass	ITO film	PDMS	Total
0.5$	0.237$	0.2$	0.937$

References

1. Zhong, J.; Liang, M.; Ai, Y. Submicron-precision particle characterization in microfluidic impedance cytometry with double differential electrodes. *Lab Chip* **2021**, *21*, 2869-2880, doi:10.1039/d1lc00481f.
2. Zhong, J.; Yang, D.; Zhou, Y.; Liang, M.; Ai, Y. Multi-frequency single cell electrical impedance measurement for label-free cell viability analysis. *Analyst* **2021**, *146*, 1848-1858, doi:10.1039/d0an02476g.
3. Tang, D.; Jiang, L.; Tang, W.; Xiang, N.; Ni, Z. Cost-effective portable microfluidic impedance cytometer for broadband impedance cell analysis based on viscoelastic focusing. *Talanta* **2022**, *242*, 123274, doi:10.1016/j.talanta.2022.123274.
